# Explorers of the twenty-first century? A systematic literature review of the scholarship on international entrepreneurs from developed economies

**DOI:** 10.1007/s11365-022-00815-4

**Published:** 2022-11-21

**Authors:** Maximilian Scheu, Andreas Kuckertz

**Affiliations:** Entrepreneurship Research Group, University of Hohenheim, Wollgrasweg 49, 70599 Stuttgart, Germany

**Keywords:** International entrepreneurship, Developed countries, Foreign entrepreneurship, Literature review, Migration

## Abstract

Throughout history, expeditionists and explorers have discovered foreign countries and new worlds, markedly influencing the lives of succeeding generations. However, as multinational enterprises have come to drive globalisation, the existence of entrepreneurial individuals without the resources of large corporations is a relatively recent phenomenon. Although research on migrant entrepreneurs demonstrates the positive impact that foreign entrepreneurial activity can have on job creation and innovation, a clear perspective on entrepreneurs from developed economies venturing abroad is lacking. The study aggregates evidence from 33 articles to establish a unifying framework that describes the foreign entrepreneurial process originating in developed economies. The framework proposes categorising foreign entrepreneurial activity according to social and economic dimensions and introduces four archetypes of foreign entrepreneurs, helping us understand the dynamics of the institutional context and the motivations for venturing into foreign environments. Finally, the study discusses the implications for foreign entrepreneurs and considers future research avenues.

## Introduction

Data from the United States and Germany suggests that foreign entrepreneurial activity accounts for as much as 20% of all venture creation (Center for American Entrepreneurship, 2017; Kollmann et al., 2020) and, therefore, plays an essential role in a country's entrepreneurial activities. For example, in Silicon Valley in the 1990s, approximately a quarter of the technology firms were co-founded by entrepreneurs with a migration background, contributing significantly to job growth, wealth creation and innovation power (Saxenian, 2000, 2002). The possible long-term effects of entrepreneurship initiated by migrants become evident when looking at the 500 most valuable American companies. Every fifth company was founded or co-founded by first- or second-generation immigrants (Center for American Entrepreneurship, 2017). Some of these entrepreneurs were migrants out of necessity, while some came from other developed economies to pursue an opportunity in the larger American market.

Much prior literature about foreign entrepreneurial activity emphasized ethnic entrepreneurship (Aldrich & Waldinger, 1990; Basu, 2006; Waldinger et al., 1990; Zhou, 2004) or migrant entrepreneurship (Baycan-Levent & Nijkamp, 2009). Most of these entrepreneurs come from less developed countries, travelling to Europe and North America to pursue their dreams of a better life and a prosperous future (International Organization for Migration (IOM), 2022). However, having difficulties entering local labour markets leaves them with no choice but to become entrepreneurially active out of necessity (Acs et al., 2008; Chrysostome, 2010).

Nevertheless, not all foreign entrepreneurs move from less developed to more developed economies. Instead, entrepreneurial movements occur in various contexts and directions (Elo, 2016; Elo et al., 2018). Scholarship on foreigners from developed economies venturing "against the tide" (e.g. Elo et al., 2019:96) has emerged, and several conspicuous start-ups founded by entrepreneurs who moved "between" developed economies (e.g. Carson & Carson, 2018; De Cock et al., 2021; March-Chordà et al., 2021) evolved (Appendix 1). These global entrepreneurs from developed countries exploit international opportunities in developed, emerging and developing nations.

Starting a business in a foreign institutional environment adds uncertainty to the venture. Entrepreneurs who take the step of venturing abroad are likely to be distinguished from those entrepreneurs in their home country. Some are "elite diasporans with developed skills and numerous alternatives for their career and livelihood" (Elo, 2016, p. 123). This specific type of entrepreneurial activity's antecedents, conditions and aims differ from the factors involved in venturing, for example, to developed countries out of necessity (Acs et al., 2008).

However, the systematic knowledge about the phenomenon is vague. Individual entrepreneurs from developed countries have not received much attention, especially not as much as the firm-level perspective of internationalizing new businesses (Dillon et al., 2020). Moreover, the multiplicity of terms within the research field of foreign venturing hampers the evolution of the academic discussion on entrepreneurs from developed economies venturing abroad and limits the theoretical exploration of the phenomenon. For example, Almor and Yeheskel (2013) called them sojourning entrepreneurs, Ngoma (2016) simply referred to foreign entrepreneurs, and Selmer et al. (2018) and Vance et al. (2016, 2017) coined the term expat-preneurs. Moreover, some studies have allocated the phenomenon to the ethnic realm (Lassalle et al., 2020; Shin, 2014; Thomas & Ong, 2015). Sometimes, the concepts and terms overlap, while at other times, they do not, leading scholars to debate the terms' applicability to real-world situations (Gruenhagen et al., 2020).

Thus, this study aims to answer the following research question: *What drives foreign entrepreneurial activity from developed economies to other developed, emerging or developing economies and with what consequences?* Employing a systematic and rigorous review of the existing literature suits the research goal of developing an omnibus perspective (Whetten, 1989) on the phenomenon. More specifically, we identified, analyzed and synthesized 33 studies providing evidence on entrepreneurs from developed economies venturing abroad.

The study provides one of the first general overviews of the foreign entrepreneurial process from developed countries to other developed, emerging or developing economies. This overview offers three significant contributions to the current literature. First, the study introduces a unifying framework for the foreign entrepreneurial process that includes the evidence from the 33 analyzed articles. Second, the article suggests exciting future research avenues that can contribute to closing the knowledge gap on the phenomenon. Hence, the academic discussion on the entrepreneurial movement from developed countries to other developed, emerging or developing economies will benefit from systematic access to international entrepreneurship research. Finally, the study proposes categorizing foreign entrepreneurial activity based on social and economic dimensions that measure the created impact. In sum, the article contributes to developing strategies to increase the impact that entrepreneurs can have on host countries. Furthermore, our proposed analytical framework is applicable independently of the different terms applied in prior research and offers necessary insights regarding foreign entrepreneurial activity.

## Review methodology

We employed a three-phase systematic literature review process (planning, conducting and reporting) (Tranfield et al., 2003) to examine the current state of knowledge. In the planning phase, the authors screened the existing literature by conducting several search rounds to identify relevant keywords, terms and articles about entrepreneurs from developed countries venturing abroad. Details of the conducting phase are presented below, along with the reporting of the findings. The review incorporated recommendations for systematic literature reviews in business and management research (Fisch & Block, 2018) and aimed to provide utmost transparency about each step undertaken (Kraus et al., 2020; Kuckertz & Block, 2021). Therefore, we followed suggestions from the *Preferred Reporting Items for Systematic reviews and Meta-Analyses* (PRISMA) (Page et al., 2021).

### Search term development

The developed search term was based on the context-intervention-mechanisms-outcomes (CIMO) framework (Denyer & Tranfield, 2009). However, applying all four steps of CIMO would have narrowed down the search too much, not allowing us to answer the research question of what drives foreign entrepreneurial activity from developed economies to other developed, emerging or developing economies and with what consequences. Therefore, the search covered both the context and the interventions while purposefully neglecting the mechanisms and outcomes. The first part of the search string approached entrepreneurship as the context of the search. The second part represented internationalisation as an intervention affecting entrepreneurship. However, the search term could not display the direction of the intervention; that is, to originate from developed economies. Therefore, we used a broad spectrum of keywords to represent the international movement describing the intervention. Table 1 provides information on each search term. The final search string was as follows:

("entrepreneu*" OR "founder" OR "founder-manager" OR "startup" OR "start-up" OR "start up" OR "venture") AND ("born global" OR "born-global" OR "foreign*" OR "international" OR "transnational" OR "trans-national" OR "expat*" OR "sojourn*" OR "ethnic" OR "diaspora*" OR "*migrant")

**Table 1:** Composition of the search terms

	**Search terms**	**Justification (search string in context)**	**Examples**
("entrepreneu*" OR "founder" OR "founder-manager" OR "startup" OR "start-up" OR "start up" OR "venture") AND ("born global" OR "born-global" OR "foreign*" OR "international" OR "transnational" OR "trans-national" OR "expat*" OR "sojourn*" OR "ethnic" OR "diaspora*" OR "*migrant")			
Entrepreneur	entrepreneu*	Includes the entrepreneur as an individual (research object) and the broader term entrepreneurship	
	founder (= founder manager) / founder-manager	Often used instead of entrepreneur No inclusion of “found*” due to too many results (> 120.000), as it includes foundation, to found (verb) and the like	
	startup / start up / start-up / venture	Included in the search term because it is the general keyword for describing new ventures and the company level within entrepreneurship research	
Internationalisation	expat*	Expatriates are foreigners working and living in a host country. Prior research has applied this term to foreigners founding a business in a host country after being expatriated to the host country by an employer	Vance et al. (2016) Selmer et al. (2018)
	foreign*	Being foreign in a country; the term describes, for example, foreign entrepreneurs founding and operating companies in China	Ngoma (2016), Kulchina (2017)
	International	International entrepreneurs are individuals identifying and pursuing opportunities across borders	Oviatt and McDougall (2005)
	Born global	Very early internationalising firms. Although the term is defined at the company level, born global adds the research stream to the results	Paul and Rosado-Serrano (2019)
	diaspora*	People who move between countries to pursue business opportunities	Elo (2016)
	sojourn*	Sojourners are, for example, Israeli entrepreneurs in China who aim to exploit opportunities, create ventures and move on to other countries	Almor and Yeheskel (2013)
	*migrant (to include immigrant or emigrant)	The term “immigrant entrepreneur” refers to migrants venturing abroad. However, the term can also be used when individuals from developed countries become entrepreneurially active in less-developed countries	Rivera-Santos et al. (2015) Abd Hamid and Everett (2021)
	transnational (or trans-national)	Established term for individuals venturing between their home countries and host countries, often drawing on synergies	Nkongolo-Bakenda and Chrysostome (2013)
	ethnic	There are intersections between the terms transnational, ethnic and migration. Thus, the inclusion of this term helped avoid overlooking relevant articles	Honig (2020)

### Article selection

We used the scientific database Scopus for article identification, as Scopus covers a broad spectrum of academic literature and is one of the most comprehensive academic databases (Gusenbauer & Haddaway, 2020). The search string was adjusted to the database search requirements. The initial search included the fields *title*, *abstract* and *keywords,* resulting in 30,106 articles before we applied inclusion and exclusion criteria. In line with previous reviews of international entrepreneurship scholarship (Peiris et al., 2012), the initial search included many different research fields. Therefore, it is plausible that the search was not limited to business-related fields but was situated at a broad analytic level to gain cross-disciplinary insights and examine several perspectives on the topic (Tranfield et al., 2003). Although this procedure produces many initial results, it limits the risk of overlooking relevant articles. Next, we iteratively applied the inclusion and exclusion criteria, narrowing down the results to the final number.

### Inclusion criteria

First, we only considered peer-reviewed English-language articles published in scientific journals to guarantee the quality of the included articles (Kraus et al., 2020). We did not include book chapters, conference papers, monographs and doctoral theses. Second, the review included articles published between 2010 and 2022. Over a decade, institutional environments change significantly in developing and emerging countries (Robinson & Acemoglu, 2012); in fact, even a country’s developmental status may change. However, covering 12 years in the search was likely to reveal relevant articles for the review. Third, the search was limited to research fields that belong to the social sciences, excluding all articles from fields belonging to the natural sciences. Lastly, applying a quality cut-off for the journal ranks ensured that the articles came from journals contributing to scientific debates (Paul & Rosado-Serrano, 2019). Therefore, journals with an explicit focus on the research area of international entrepreneurship served as a quality reference. After scoping the journals, we decided to apply a quality cut-off equal to the Scopus CiteScore of 3.0. This procedure included one-third of the journals from the Business, Management, and Accounting research field (Scopus, 2022), limiting the review to articles that contribute to ongoing scientific conversations and ensuring the inclusion of the most influential target-field journals. The procedure resulted in an initial sample of 2,368 articles.

### Exclusion criteria

Most of the identified 2,368 articles belonged to international business, entrepreneurship and international marketing research areas. This confirmed that the search string and inclusion criteria functioned properly, thus allowing us to identify an ample number of articles for review. We screened the identified articles by first reading the titles and abstracts and excluding irrelevant studies (Booth et al., 2021) by applying the exclusion criteria described below.

First, the object of analysis was entrepreneurs at the individual level. Therefore, articles investigating the firm level – for example, as in the case of the internationalisation of a new venture– were excluded. Second, only studies that clearly emphasised foreign entrepreneurs instead of local entrepreneurs fit the research goal. Third, we excluded all studies investigating entrepreneurs from non-developed economies. However, it was difficult to categorise countries accurately according to their development level, given that.

“there is no established convention for the designation of “developed” and “developing” countries or areas in the United Nations system. In common practice, Japan in Asia, Canada and the United States in northern America, Australia and New Zealand in Oceania, and Europe are considered “developed” regions.” (United Nations, 2003; see also Organization for Economic Co-operation and Development, 2006)

This definition by the United Nations dates back to 2003. It shows that no clear indicators for such categorisation exist. Therefore, we decided to rely on two parameters instead of a single source. First, the Inequality-Adjusted Human Development Index (IHDI) considers “very high human development nations” to have an IHDI equal to or higher than 0.8 (United Nations, 2020). Taking life expectancy, years of schooling and gross national income per capita into account, the IHDI is an adequate measurement of a country’s stage of development. Second, the high-income-nations list provided by the Organisation for Economic Co-operation and Development (OECD, 2022) was useful in classifying nations’ development (Karanikolos et al., 2016). Comparing IHDI and the list, most of the countries overlap. However, some highly developed IHDI countries are not part of the OECD’s list. Therefore, we introduced the United Nations Human Development Index (United Nations, 2020) value of over 0.85 as the third criterion and triangulated the list by excluding nations that did not fulfil at least two of the three criteria. Table 2 provides information on countries meeting the following criteria:

**Table 2:** Developed countries composed of OECD High Income Nations list, United Nations’ HDI and IHDI lists

HDI & IHDI according to United Nations (2019)			**OECD member states**	**Meeting at least two criteria for a “developed” country and thus included in the review**
**Country**	**IHDI**	**HDI**		
Very high human developed				
Norway	0.899	0.957	Yes	Norway
Iceland	0.894	0.949	Yes	Iceland
Switzerland	0.889	0.955	Yes	Switzerland
Finland	0.888	0.938	Yes	Finland
Ireland	0.885	0.955	Yes	Ireland
Denmark	0.883	0.940	Yes	Denmark
Sweden	0.882	0.945	Yes	Sweden
Netherlands	0.878	0.944	Yes	Netherlands
Slovenia	0.875	0.917	Yes	Slovenia
Germany	0.869	0.947	Yes	Germany
Australia	0.867	0.944	Yes	Australia
Czech Republic	0.860	0.900	Yes	Czech Republic
Belgium	0.859	0.931	Yes	Belgium
New Zealand	0.859	0.931	Yes	New Zealand
Austria	0.857	0.922	Yes	Austria
United Kingdom	0.856	0.932	Yes	United Kingdom
Canada	0.848	0.929	Yes	Canada
Japan	0.843	0.919	Yes	Japan
Estonia	0.829	0.882	Yes	Estonia
Luxembourg	0.826	0.916	Yes	Luxembourg
Hong Kong	0.824	0.949	No	Hong Kong
Malta	0.823	0.895	No	Malta
France	0.820	0.901	Yes	France
South Korea	0.815	0.916	Yes	South Korea
Israel	0.814	0.919	Yes	Israel
Poland	0.813	0.880	Yes	Poland
Singapore	0.813	0.938	No	Singapore
United States	0.808	0.926	Yes	United States
Slovakia	0.807	0.860	Yes	Slovakia
Cyprus	0.805	0.887	No	Cyprus
High Human developed				
Greece	0.791	0.888	Yes	Greece
Hungary	0.791	0.854	Yes	Hungary
Lithuania	0.791	0.882	Yes	Lithuania
Italy	0.783	0.892	Yes	Italy
Croatia	0.783	0.851	No	
Latvia	0.783	0.866	Yes	Latvia
Spain	0.783	0.904	Yes	Spain
Belarus	0.771	0.823	No	
Kazakhstan	0.766	0.825	No	
Portugal	0.761	0.850	Yes	Portugal
…	…	…	…	
Chile	0.709	0.851	Yes	Chile
Costa Rica	0.661	0.810	Yes	
Turkey	0.683	0.820	Yes	
Mexico	0.613	0.779	Yes	
Colombia	0.595	0.767	Yes	

$$\begin{aligned}(\mathrm{IHDI}>=& 0.8\;\mathrm{AND\;HDI}>= 0.85)\;\mathrm{OR}\;(\mathrm{IHDI}>= 0.8\;\mathrm{AND\;OECD\;Member})\;\mathrm{OR}\\&(\mathrm{HDI}>= 0.85\;\mathrm{AND\;OECD\;Member})\end{aligned}$$

Screening the titles and abstracts, the lead author sorted the articles into two groups according to their relevance or irrelevance to the review. Articles deemed uncertain after reading the title and abstract were added to the third group of articles that require further analysis. Next, a research assistant used a sub-sample of 200 randomly chosen abstracts to triangulate the applicability of the research team’s exclusion criteria. Most of the articles were classified identically after reading the title and abstract. However, the articles whose classification was ambiguous were added to the group considered to require further analysis. The authors read all articles that were identified to be included or classified to be ambiguous entirely to not exclude any relevant articles. Therefore, the exclusion criteria were suitable for initially sorting out the abstracts. The final decision between inclusion and exclusion in the review was made only after thoroughly reading the articles that were ambiguous or identified to be relevant after the abstract screening. Thus, although some articles fulfilled the initial criterion of dealing with foreigners venturing abroad, we excluded the articles that did not add value to the review – for example, articles that investigated entrepreneurial activities in the past centuries (e.g. Lopes et al., 2018; Sifneos, 2010). However, each exclusion from the review was discussed extensively to avoid overlooking relevant articles.

**Fig. 1:**
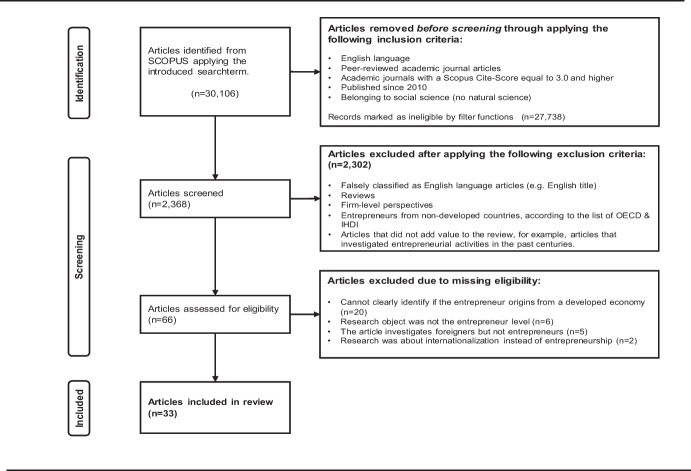
Search procedure for article identification following the PRISMA framework

This procedure resulted in the final sample of 33 articles. Figure 1 summarises the search and selection procedure following the recommended PRISMA-process (Page et al., 2021; Stovold et al., 2014); the amplified PRISMA checklist is in Appendix 2.

### Analysis

Booth et al. (2021, p. 73) recommended using analysis software for qualitative literature synthesis. We used MAXQDA 2020 (version 20.0.1b) to analyse and code the articles. Furthermore, we employed a data extraction sheet (Booth et al., 2021) to collect relevant information. Table 3 provides key information from the data extraction sheet and the reviewed articles, including the methodologies, research question, entrepreneurs’ countries of origin and residence, and a summary of each reviewed article. When reading, analysing and coding the identified articles, the research team considered the following research question: What drives foreign entrepreneurial activity from developed economies to other developed, emerging or developing economies and with what consequences?

**Table 3:** Overview of the reviewed articles

**#**	**Author (year)**	**Research question**	**Methodology**	**Data source**	**CoO**	**CoR**	**Key findings**
1	Chrysostome (2010)	Classification of and distinction between necessity-driven and opportunity-driven immigrant entrepreneurs	Conceptual	No primary data; derived from the literature	N/A	N/A	The article suggests five types of survival factors for necessity-driven immigrant entrepreneurs: ethnocultural factors, financial factors, managerial factors, psycho-behavioural factors and institutional factors
2	Hudnut and DeTienne (2010)	*Not directly reported. However, the described case entrepreneurs searched for answers to the following questions:* (1) how to develop a product that could be used in countries around the world; (2) how to design a business model that would enable them to sell products to customers at the base of pyramid markets and meet their triple-bottom-line objectives; and (3) how they should develop their management teams and key partnerships	Qualitative (case study)	Interviews and secondary data	United States	Philippines / South East Asia	The article describes the retrospective case of Envirofit, providing insights into a foreign venture aiming for the triple bottom line in an emerging country
3	Connelly (2010)	*Not directly reported:* What is an expatriate, and how can the phenomenon of expatriatism be conceptualized?	Quantitative	Survey of 160 expatriates	Various	Various	The literature has not examined the phenomenon of expatriates enough, whose dimensions have changed over time. The author categorises expatriates into (1) traditional expatriates, (2) transnational entrepreneurs, (3) ambassadors and (4) world-changing entrepreneurs
4	Yang et al. (2011)	How do biological kinship and the biological theories of altruism influence the behavior of ethnic entrepreneurs?	Quantitative	A survey of Korean entrepreneurs in the United States	South Korea	United States	In line with inclusive fitness theory, the study found that Korean “immigrant entrepreneurs are more likely to perceive kin as more trustworthy and assist them more frequently than non-kin” (p. 642). Thus, foreign entrepreneurs are likely to rely more on their kin than on other kinds of associates
5	Marshall (2011)	What are the distinguishing characteristics of for-profit social entrepreneurs regarding markets as arenas for confronting challenging social issue?	Qualitative (case study)	Case-related data	United States / United States–Brazil	Guatemala and Nicaragua, Indonesia, Argentina	This study provides a first conceptualisation of international for-profit social entrepreneurs (IFPSEs) as a sub-group of international entrepreneurs. The study found that (1) IFPSEs are “committed to a global social issue,” (2) the market can address social issues, (3) consumers must be connected to a company’s social mission, (4) deep knowledge about culture, society and ecosystems is crucial and (5) partnerships matter
6	Ensign and Robinson (2011)		Conceptual (with an example of “South to North immigration” that can be overlooked)	N/A	Canada	N/A	Immigrant entrepreneurs usually start by operating in a niche market. Then, their “products, processes, and ideas [started] being adopted and gaining status as ‘normal’” (p. 48) Therefore, immigrant entrepreneurs launch innovations from their niche markets into local mainstream markets
7	Almor and Yeheskel (2013)	*None: Grounded theory in search of a conceptual model for entrepreneurs from developed countries*	Qualitative grounded theory (case study)	Interviews	Israel	China	Sojourning entrepreneurs are entrepreneurs with a unique combination of know-how, knowledge and experience gained in developed countries who aim to exploit opportunities in emerging and developing countries. They do not assimilate into CoRs because they want to exploit opportunities globally. Therefore, they do not intend to stay in one place for a long time
8	Nkongolo-Bakenda and Chrysostome (2013)	*Not directly reported:* What are the determinants of a diaspora’s international new venture creation in a country of origin?	Conceptual	N/A	N/A	N/A	The study proposes a theoretical framework of determinants for diaspora entrepreneurship based on international business, international entrepreneurship and entrepreneurship theories
9	Storti (2014)	How do network structure and content affect the entrepreneurial process?	Qualitative (case study)	Interviews	Italy	Germany	Although the investigated cases had the same ethnic background, their modes of international entrepreneurship differed in leveraging networks and co-ethnics, hiring and branding. Thus, when investigating international entrepreneurship, a multi-level view should be considered
10	Shin (2014)	How do Korean entrepreneurs in Kansas City interact with and adjust to their immigrant business community in ethnic and local contexts?	Qualitative	Interviews	South Korea	United States	Over time, immigrant entrepreneurs’ business numbers and types have changed. Moreover, as the wealth of the home country increases, immigrant entrepreneurs must adopt a transnational lens in the host country to leverage transnational opportunities in the home country to develop their businesses
11	Knight (2015)	What factors motivated Polish ethnic entrepreneurs’ actions during their migration period?	Qualitative grounded theory	Interviews	Poland	Wales	Immigrant entrepreneurs motivations for starting a business change over time. It is a dynamic process affected by several components. While opportunity was the main driver from the entrepreneurs’ perspectives, it seems that an underlying dissatisfaction with their embeddedness was a hidden motive for many entrepreneurs
12	Heidenreich et al. (2015)	How do the use of political strategies and entrepreneurial overconfidence individually and jointly affect entrepreneurial decision-making when investing and operating in highly uncertain environments?	Qualitative (single case study)	Interviews, secondary data on the company, industry and host country from multiple sources	Germany	Ghana	Cognitive biases influence the market entry strategies and post-entry strategies of foreign entrepreneurs. For example, entrepreneurial overconfidence is a factor once an entrepreneur is convicned by an idea. When focusing on an opportunity entrepreneurial overconfidence hinders a rational and critical evaluation of foreign market opportunities due to the entrepreneur’s cognitive biases
13	Chandra et al. (2015)	How do entrepreneurs recognise and pursue opportunities in international markets over time? To what extent, if any, can we use the opportunity-portfolio perspective to explain this process?	Qualitative (single case study)	Interviews and secondary data	New Zealand	Australia	Based on their longitudinal in-depth case study, the authors introduced the concept of “opportunity portfolio processing” (p. 221). Their framework accounts for opportunity sets from the creation to the testing of opportunities
14	Thomas and Ong (2015)	How did Korean dry cleaners in Southern California use ethnic resources to politically mobilise in response to an environmental policy targeted at their industry?	Mixed methods	Interviews, secondary data, survey	South Korea	United States	Korean immigrant entrepreneurs used ethnic networks to mobilise. Thus, organised ethnic associations foster and support their ethnic entrepreneurial communities by mitigating cultural barriers and bundling individuals’ impacts
15	Elo (2016)	What makes people become entrepreneurs in a transition economy like Uzbekistan, especially if they have other alternatives to prosper elsewhere?	Qualitative (case study)	Longitudinal interviews	Uzbekistan, Bukhara Israel, Korea, Tajikistan and Germany	Uzbekistan	Insights into diaspora entrepreneurship at the individual level and determinants of venturing into transitional economies with a developing institutional environment
16	Lassalle and McElwee (2016)	*Not directly reported:* What are the dimensions and components of the opportunity structures of ethnic minority entrepreneurs?	Qualitative	Interviews	Poland	Scotland	The article emphasises the crucial role of the co-ethnic society in rolling out a business and the importance of co-ethnics and family ties in business decisions. Therefore, it introduces a new tool for performing systematic comparative analyses of ethnic minority entrepreneurs’ opportunity structures in relation to different influential factors that affect founding and business decisions
17	Ngoma (2016)	How do foreign entrepreneurs, as outsiders, establish and develop close *guanxi* relationships as they set up and operate their Shanghai-based SMEs?	Qualitative	Interviews, triangulated by survey data	Various	China	Foreign entrepreneurs in China draw on the local network relationship theme called “guanxi.” They use guanxi to refer to relationships and connections that are beneficial to their businesses and can even become a means of survival. However, the adaptation of business manners and cultural attitudes by foreigners differs from local entrepreneurs
18	Lassalle and Scott (2018)	*Not directly reported:* What are the breakout strategies of migrant entrepreneurs during their business development process?	Qualitative	Interviews and field observations	Poland	Scotland	The article extends our knowledge migrant entrepreneurs’ diversification strategies during the business development process and focuses on the spatial, social and institutional dimensions
19	Lundberg and Rehnfors (2018)	*Not directly reported:* What types of opportunities do transnational entrepreneurs identify, and how do institutional and cultural contexts impact these opportunities?	Qualitative	Interviews	Nordic (Sweden and Finland)	Hong Kong	Transnational entrepreneurs are more opportunity driven than necessity driven. They exploit opportunities using their social and human capital and experiences in the CoOs and CoRs
20	McHenry and Welch (2018)	Are Western immigrants similar to either self-initiated expatriates or host country nationals, or do they form a discrete sub-group?	Qualitative (case study)	Interviews	Various (France, Germany, Denmark, Canada, Australia etc.)	Vietnam	The study analyses the black box of Western immigrant entrepreneurs in emerging countries. It iteratively analyses why Western expatriates become entrepreneurs and the kinds of businesses they engage in. This study extends our knowledge of expatriate entrepreneurship from developed to less developed countries and its preconditions and motivations
21	Selmer et al. (2018)	Are personal characteristics of expat-preneurs different from those of company employed Self-Initiated Expats?	Quantitative	Survey data	Various	China, Hong Kong, Singapore	Expat-preneurs’ specific personal characteristics differ from those of Self-Initiated Expats (age, position, time in current job in host location, time as expatriate, time in host location), suggesting that the time spent in the host country has a significant impact on being an entrepreneur
22	Carson and Carson (2018)	How do international lifestyle-driven migrant entrepreneurs contribute to tourism development and the functioning of interactive tourism innovation systems in a sparsely populated region of northern Sweden? 1) What were the motivational and mobility characteristics of these immigrant entrepreneurs? 2) How have they contributed to the accumulation of local capitals? 3) How have they interacted with local stakeholders to stimulate knowledge sharing, learning, collaboration and innovation spillovers at the local system level?	Qualitative (case study)	Interviews	Various (Germany, France, the Netherlands, UK, etc.)	Sweden	Lifestyle entrepreneurs in the tourism sector bring innovation and new resources (e.g. language) to the host country by exploiting the market opportunities that locals do not see or do not pursue. In addition, they use their cultural and social capital to establish strong ties within the foreign community
23	Elo et al. (2019)	What attracts Jewish diaspora entrepreneurs or nascent entrepreneurs to certain locations (against the tide in terms of migration)? How and why do they decide to migrate?	Qualitative	Interviews	Various; all Jewish	China, Central Asia	The motivation to become an entrepreneurial actor “against the tide” depends on pull and push factors. Interestingly, some determinants that appear to negatively impact foreign entrepreneurship, such as institutional voids, seem to attract some foreigners as they enjoy first-mover advantages
24	Lassalle et al. (2020)	What is the dynamic interplay between processes of network proximation/distanciation and opportunity creation in migrant entrepreneurship?	Qualitative	Interviews	Poland	United Kingdom	Migrant entrepreneurs move in three types of networks: 1) origin country networks 2) host country migrant networks and 3) host country indigenous networks The findings show that the embeddedness of migrant entrepreneurs involves multiple dimensions: relational (professional connections within business networks), social (socialisation and community ties) and structural (formal and informal relations with institutions) (p. 525)
25	De Cock et al. (2021)	How does founders’ international experience (or lack thereof) affect the strategic decisions taken regarding their ventures’ internationalisation process?	Qualitative	Interviews and secondary data	Belgium	United States (mainly Silicon Valley, some in multiple countries)	Building on imprinting theory, the study identified four types of international entrepreneurs whose internationalisation motives derived from their previous international experience and beliefs:multi-ecosystem leveragers, ecosystem accelerators, ecosystem grownups and ecosystem adventurers
26	Dillon et al. (2020)	1) How has the experiencing process changed for entrepreneurs operating at the interface of physical and digital environments within international entrepreneurial firms? 2) How do the international experiences of entrepreneurs operating at the interface of digital and physical business environments influence the emergence of new business ideas?	Qualitative (case study)	N/A	Multiple (Israel, France, Australia, New Zealand, Malaysia, Chile)	Australia	Digital technologies support international opportunity recognition by increasing opportunity confidence. However, foreign entrepreneurs cannot neglect traditional forms of international opportunity recognition
27	Eimermann and Kordel (2018)	How do the studied lifestyle migrants’ ongoing quests for a better life and their evolving mix of embeddedness in the studied New Immigrant Destinations affect each other?	Qualitative	Interviews	Multiple (the Netherlands, Canada, Germany, Denmark, UK, etc.)	Slovenia and Sweden	Lifestyle entrepreneurs in new immigrant destinations contribute to local developments based on their backgrounds. In addition, their aspirations influence how and why they use institutional assistance and support. The study indicated that lifestyle entrepreneurs can become embedded in both co-ethnic and local groups, depending on the business and area
28	Gurău et al. (2020)	How and when do Transnational Entrepreneurs (TEs) access and accumulate various types of resources during their personal and professional evolution? What are the main elements of TEs’ entrepreneurial habitus? How do TEs use their resources and habitus to exploit transnational opportunities through various boundary-spanning activities?	Qualitative	N/A	Israel	China	The study proposed a model of transnational entrepreneurship that combines practice theory with boundary-spanning activities for transnational entrepreneurs. The authors described three phases of the transnational entrepreneurial journey: 1. early acculturation and life experiences; 2. professional experience and expatriation; and 3. entrepreneurial activities
29	March-Chordà et al. (2021)	What locational factors are most important to immigrant entrepreneurs and should therefore be prioritised and promoted by entrepreneurial ecosystems in their strategy to attract foreign entrepreneurs? How important are locational factors in the future development of startups?	Qualitative comparative analysis	Interviews	Spain	United States	The study outlined the locational factors that foreign entrepreneurs should be most aware of when arriving in top entrepreneurial ecosystems. The study found the “image of Silicon Valley” to be essential for Spanish entrepreneurs. Furthermore, access to investors and the absence of a certain level of human resources positively impact foreign entrepreneurs
30	Abd Hamid and Everett (2021)	How do migrant entrepreneurs from a more developed home country use coethnic ties in a relatively less developed host country?	Qualitative (case study)	N/A	South Korea	Malaysia	The home country’s development level influences how co-ethnic ties in the host country are mobilised and structured. For example, the study found that the Korean co-ethnic community was more organised than other national enclaves and contained many official institutions to ensure Korean well-being in the host country
31	Tucker and Croom (2021)	Why do some social entrepreneurs choose to assume risks, financial and otherwise, to venture for foreigners?	Conceptual	N/A	N/A	N/A	The authors posed the overarching question of why some entrepreneurs venture for foreigners. This is an important concept in the realm of social entrepreneurship (e.g. contributing to the development of countries). The study describes social class logic, religious logic and multicultural experiences as drivers of social entrepreneurship for foreigners
32	Kumpikaitė-Valiūnienė et al. (2021)	Do the demographic characteristics and motivations to expatriate of pre-departure and transnational self-initiated expat-preneurs differ?	Quantitative	Survey data	Lithuania	Various	Gender, age and education level differed between pre-departure expat-preneurs and already transitioned expat-preneurs who had started their companies abroad
33	Goxe et al. (2022)	*Not directly reported:* (1) What are the characteristics of nascent international entrepreneurs? (2) How can social networks strengthen or weaken the entrepreneurs’ internationalisation path?	Qualitative	Interviews and secondary data	France	China	The study helps to understand “liabilities of outsidership” by evaluating foreign entrepreneurs’ networking behaviour by drawing on Bourdieu’s theory of practice It identifies two types of foreign entrepreneurs: First, the “Fallen Icaruses” who cannot adjust to a global habitus and penetrate host-country networks. Second, the “Global Argonauts” who adopt a global habitus that enables them to penetrate networks in the host country and to pursue the opportunities facilitated their expanded habitus

*CoR* Country of Residence, *CoO* Country of Origin

## Results

### Descriptive results

We used the bibliographic data from the selected articles (*n* = 33) to run a bibliographic coupling network analysis using the *VOSviewer* software (Van Eck & Waltman, 2010). The *VOSviewer* software applies an algorithm that visualizes similarities on a distance-based approach (Van Eck & Waltman, 2014) and has proven a reliable tool for bibliometric coupling analysis (Cobo et al., 2011). The algorithm places items according to their relatedness. Closely related items are nearer to one another; an increasing distance implies weaker relations between the nodes (Waltman et al., 2010). The strongest related items within the network are placed in the centre, while weaker connections are somewhat at the edge.

**Fig. 2:**
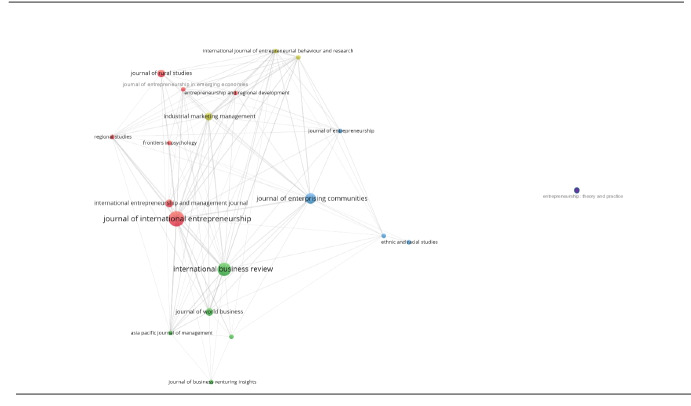
Journal map displaying the bibliographic coupling of the sources (Entrepreneurship theory and practice (cluster 5) shows no connection to the other articles, leading to a great distance in the journal map. For visibility reasons, it is not included in the figure)

Figure 2 displays the journal map based on bibliographic couplings of the 33 articles' sources (Appendix 3 contains bibliographic summary statistics; Appendix 4 shows the document map including the citation count of the studies). The selected articles were published in various fields and journals, indicating a cross-sectoral audience. Each circle represents one of the journals; the size indicates the number of published articles (increasing size with an increasing number of articles). The colour scheme allocates the journals to a cluster (see Table 4) following the VOSviewer integrated clustering method based on association strengths' relatedness (Waltman et al., 2010) with a resolution of 1.0. Table 4 provides the journal source, their allocated cluster, and the number of articles. Most journals were business related and focused on entrepreneurship.

**Table 4:** Journals allocated to the clusters

**Cluster 1 (red)**	**Cluster 2 (green)**	**Cluster 3 (blue)**	**Cluster 4 (yellow)**	**Cluster 5 (not in** **Fig.** 2**due to high distance)**
Journal of International Entrepreneurship (*n* = 5)	International Business Review (*n* = 4)	Journal of Enterprising Communities (*n* = 3)	Industrial Marketing Management (*n* = 1)	Entrepreneurship Theory and Practice (*n* = 1)
International Entrepreneurship and Management Journal (*n* = 2)	Journal of World Business (*n* = 2)	Journal of Entrepreneurship (*n* = 1)	International Journal of Entrepreneurial Behaviour and Research (*n* = 1)	
Journal of Rural Studies (*n* = 2)	Journal of Business Ethics (*n* = 1)	Thunderbird International Business Review (*n* = 1)	Journal of Ethnic and Migration Studies (*n* = 1)	
Frontiers in Psychology (*n* = 1)	Asia Pacific Journal of Management (*n* = 1)	Ethnic and Racial Studies (*n* = 1)		
Journal of Entrepreneurship in Emerging Economies (*n* = 1)	Journal of Business Venturing Insights (*n* = 1)			
Regional Studies (*n* = 1)	International Marketing Marketing Management (*n* = 1)			
Entrepreneurship Regional Development (*n* = 1)				

Method = association strengths, Clustering resolution = 1.0

The *Journal of International Entrepreneurship* (red), *International Business Review* (green), and also the *Journal of Enterprising Communities* (blue) built three major clusters and published most about foreign entrepreneurs from developed countries. The analysis further shows that journals with an international (entrepreneurship & business) focus published most content on foreign entrepreneurship originating from developed countries. One article published in *Entrepreneurship: Theory and Practice* (Hudnut & DeTienne, 2010; see Cluster 5) has no linkages with the other articles and, therefore, is distanced from the connected journal map.

International entrepreneurship research entails intersections with various research disciplines such as anthropology, sociology, international business and economics, to name a few (Oviatt & McDougall, 2005). Furthermore, most studies describe the phenomenon of foreigners venturing abroad and provide little theoretical explanation for the determinants, antecedents, and consequences typically discussed in superordinate management journals. The theories developed in the Western context do not necessarily apply to emerging and developing countries (Wright et al., 2005). Therefore, familiarising oneself with the phenomenon and then applying, testing and developing theories is plausible.

However, the small number of quantitative studies and the lack of theoretical explanatory power indicate that access to entrepreneurs from developed countries venturing abroad is limited. Not reaching a critical mass of entrepreneurs operating in comparable environments leads to a lack of quantifiable results, which, in turn, results in the inability to test theories. Consequently, the theoretical lens is not sharpened, scholars do not apply the same theories to explain the phenomenon more deeply, and there are no ongoing discussions on an agreed set of issues or theories within or across journals.

**Fig. 3:**
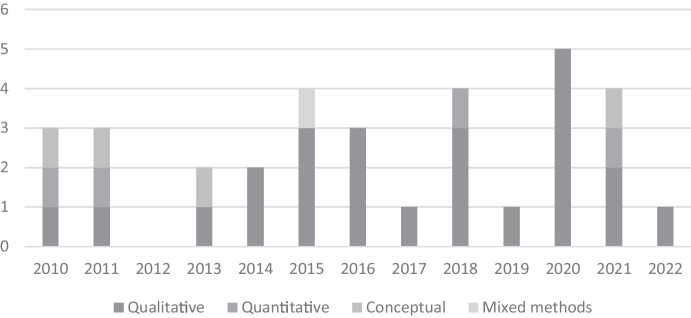
Timeline and methodological designs of the reviewed articles

Figure 3 displays the methodological designs of the reviewed articles. Many authors have applied qualitative research designs (24), drawing mainly on case studies. Accordingly, interviews were the most common primary data source. At the same time, only a minority of the studies collected and investigated quantitative data (4) (Connelly, 2010; Kumpikaitė-Valiūnienė et al., 2021; Selmer et al., 2018; Yang et al., 2011), and few conceptual studies (4) (Chrysostome, 2010; Ensign & Robinson, 2011; Nkongolo-Bakenda & Chrysostome, 2013; Tucker & Croom, 2021) contributed to developing the understanding of the field and its boundaries.

Most studies have focused on entrepreneurial activities between developed countries (15), followed by a developed-to-less-developed perspective (10). By contrast, only one study investigated developed-to-least-developed countries. The most frequent countries of residency (CoR) were China and the US. However, it was surprising that little to no research focused on emerging economies, such as India (none), or fast-developing countries in Africa (only 1 study). One could imagine that these environments offer many possibilities to entrepreneurial foreigners from developed countries in terms of social development (Rivera-Santos et al., 2015; Tucker & Croom, 2021) and economic opportunities, especially within Africa (Heidenreich et al., 2015). Therefore, the minimal emphasis on Africa, the demographically fastest-growing continent with great economic potential, was unexpected. Table 5 shows the movements from the entrepreneurs’ country of origin (CoO) to their CoR.

**Table 5:** Countries of Origins (CoR) and Countries of Residency (CoR)

**CoO /** **CoR**	AU	BEL	CAN	Chile	DEN	FND	FRA	GER	Israel	Italy	LIT	NLD	NZ	POL	SKOR	Spain	SWED	US	UK
Australia	X			X			X		X				X						
China							X		X										
Hong Kong						X											X		
Germany										X									
Ghana								X											
Guatemala																		X	
Indonesia																		X	
Malaysia															X				
Nicaragua																		X	
Phillippines																		X	
Scottland														X					
Slovenia			X	X				X				X							
Singapore																			
Sweden			X	X			X	X				X							X
UK														X					
US		X													X				
Uzbekistan								X	X										
Vietnam			X				X	X											
Wales														X	X				

### Towards a unifying framework

**Fig. 4:**
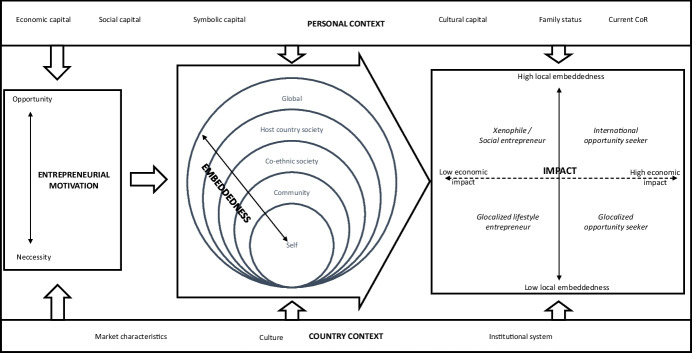
Unifying framework

The guiding research question of what drives foreign entrepreneurial activity from developed economies and with what consequences was complex and multi-faceted. Unsurprisingly, the relevant articles were heterogeneous. Based on the reviewed articles, Fig. 4 synthesises the evidence into one overarching framework that furthers our understanding of the phenomenon. The following paragraphs will discuss the elements of the framework and how they affect the foreign entrepreneurial journey.

Starting with contextual effects, the framework shows that external and internal factors, namely the entrepreneur’s context and the country’s context, play a significant role (e.g. Chrysostome, 2010; Lassalle & Scott, 2018) throughout the entrepreneurial process. Contextual factors matter in entrepreneurship (Welter, 2011). Therefore, the context impacts how entrepreneurs discover opportunities, how they embed themselves in a foreign society and what the impacts of their endeavours are. Next, the framework emphasises the antecedent circumstances and motivations of foreign entrepreneurs (e.g. Carson & Carson, 2018; Chandra et al., 2015; Knight, 2015) that trigger the entrepreneurial process abroad and explains how these factors translate into embeddedness in the host country (e.g. Lassalle & McElwee, 2016; Storti, 2014). Finally, the framework analyses how entrepreneurs impact host countries (e.g. Goxe et al., 2022; McHenry & Welch, 2018).

The framework was constructed after the first round of analysis and helped arrange the articles into an overarching structure. The framework’s elements were underpinned by the findings from the analysis of the articles. Therefore, we went back and forth between the literature and the framework.

### Influential factors

It is well known that entrepreneurship is strongly shaped and influenced by the context in which it is practised (Welter, 2011). Therefore, adapting to external circumstances, particularly to the spatial context, cultural context and institutional framework, is critical for foreign entrepreneurs (e.g. Elo, 2016). Furthermore, foreign entrepreneurs’ personal circumstances impact the entrepreneurial journey abroad (e.g. McHenry & Welch, 2018). Our framework focuses on two overarching contextual dimensions. First, it addresses entrepreneurs’ personal dimension. Drawing on Whetten’s (1989) work, the personal dimension, or the *who* dimension, is important. Therefore, the entrepreneurs’ family status, prior experiences, and networks and skills matter. Second, the country context, or the *where* dimension, addresses various aspects of the host country, such as culture, institutional framework, and market opportunities (Welter, 2011; Whetten, 1989).

#### Personal context

Scholars have shown little consistency in applying theoretical frameworks to describe the personal contexts of foreign entrepreneurs. For example, Drori et al. (2009) and Goxe et al. (2022) drew on Bourdieu (1990) work on the different forms of personal capital and cultural fields. When an entrepreneur operates in a cross-cultural setting, his fields should adapt to the new “global economic field” (Bourdieu, 2005, p. 229). Regardless of the underlying theoretical lens, integration in the host-country context facilitates or complicates entrepreneurial work depending on entrepreneurs’ skills and resources, affecting their overall entrepreneurial activities on many levels.

Entrepreneurs’ resources, such as financial capital, managerial skills, network ties (Chrysostome, 2010) and prior experiences abroad, influence their capacity to recognise opportunities (Lundberg & Rehnfors, 2018), the partners with whom the entrepreneurs interact (Ngoma, 2016) and how they embed themselves within the host country (Gurău et al., 2020). Furthermore, the personal characteristics involved in adapting to new environments and expanding one’s resources, such as having a growth-oriented mindset, increase the likelihood of succeeding in the venture. By adopting a “dual resource system,” entrepreneurs first draw on their skills and resources before actively seeking to increase their resource endowments throughout the CoR society (Gurău et al., 2020).

Furthermore, the entrepreneurs’ personal context affects their embeddedness in various ways. For example, having local family ties eases movement within local institutional environments (McHenry & Welch, 2018). Loose contacts with host country nationals and difficulties accessing host country institutions lead to foreign entrepreneurs operating in co-ethnic niches (Lassalle & Scott, 2018). Establishing networks with other foreign entrepreneurs is helpful and easier to accomplish than attaining access to locals (Carson & Carson, 2018). Such networks help balance the lack of knowledge and difficulties in interacting with local institutions (Lassalle & McElwee, 2016). However, to achieve greater impact, entering local or global mainstream markets is essential (Ensign & Robinson, 2011; Lassalle et al., 2020).

Moreover, the personal context is not limited to entrepreneurs’ resources; rather, it affects various dimensions of their lives and the whole entrepreneurial journey. For example, the desire to live in a particular place is one motivation that drives some entrepreneurs from developed countries to venture abroad (e.g. Carson & Carson, 2018; Eimermann & Kordel, 2018). Therefore, personal factors can initiate entrepreneurial activity in a foreign country (e.g. McHenry & Welch, 2018; Selmer et al., 2018) or restrain an entrepreneur from being fully embedded within a foreign context as “mixed marriages and generational status influence the willingness to migrate to a new location” (Elo et al., 2019, p. 99).

#### Country context

The country context influences the entrepreneurial journey in several ways (Welter, 2011). The institutional environment affects entrepreneurs and the founding of their companies (North, 1990). Entrepreneurs who venture into foreign CoRs operate in spatial dimensions different from those of their CoOs. Institutional support and familiarity with the institutional environment are limited (Lassalle & McElwee, 2016), while institutional voids lead to restrictions and denied access, whether due to cultural distance or limited market knowledge (Marshall, 2011) – these issues are known as liabilities of foreignness (Hymer, 1976; Zaheer, 1995).

Although coming from developed countries and, on average, enjoying good education (e.g. Almor & Yeheskel, 2013; Carson & Carson, 2018; Goxe et al., 2022; Gurău et al., 2020; Selmer et al., 2018; Thomas & Ong, 2015), foreign entrepreneurs face challenges related to the local institutional settings of their CoRs. Therefore, it makes little difference whether they operate in another developed country (e.g. Eimermann & Kordel, 2018; Thomas & Ong, 2015) or in an emerging market (e.g. Elo, 2016) where institutional voids are expected (Wright et al., 2005). When venturing abroad, the starting point is to understand the emotions of local people (Elo, 2016). The entrepreneurs' limited knowledge about the host country's institutional environment increases the risk of failing the business (Heidenreich et al., 2015)..

However, the uncertainty of the environment encourages some entrepreneurs (Elo et al., 2019) to venture abroad and strengthens the co-ethnic networks of those who dare to do so (Shin, 2014; Yang et al., 2011). Thus, the host country’s institutional environment affects all steps along the entrepreneurial journey, starting from opportunity creation and entrepreneur embeddedness, and determines the social and economic impact that an entrepreneur can create.

### Motivation

Knight (2015) underscored the complexity of founding a startup and suggested that the clear difference between necessity-driven and opportunity-driven (Block & Wagner, 2010) motives may sometimes be misleading. Nevertheless, the literature often associates migrant entrepreneurs moving from less-developed to more-developed countries with necessity-driven entrepreneurship (Chrysostome, 2010), compared to entrepreneurs from developed countries, who are said to act based on opportunity (Acs et al., 2008).

However, some entrepreneurs from developed countries venturing abroad do so out of necessity as well. For example, McHenry and Welch (2018, p. 5) found that the need to remain in the host country is a sufficient motivation for setting up a company. Unsurprisingly, the need to stay in a specific place depends on social connections, such as family ties in the CoR, which influence entrepreneurial decisions (Elo et al., 2019, p. 101). Indeed, foreigners’ “personal circumstances and social relationships often support the [founding] decision” (Selmer et al., 2018, p. 141). Moreover, entry barriers to the local labour market arise independently of individuals’ education (Knight, 2015) and apply to foreigners from developed countries as well. Challenges such as language barriers (Thomas & Ong, 2015) paired with differing salary expectations (McHenry & Welch, 2018) or job dissatisfaction issues (Storti, 2014) drive foreigners towards entrepreneurial activity. Therefore, although the particular motive for venturing abroad differs among individuals, for some entrepreneurs, the motivation for founding a business has to do with satisfying the need to stay in the CoR.

The decision to pursue a particular lifestyle is somewhere between chasing an opportunity and acting entrepreneurially out of necessity (Eimermann & Kordel, 2018; Carson & Carson, 2018). The question arises regarding which end of the continuum these entrepreneurs belong to. On the one hand, being unable to live a desired way of life in the CoO creates the pressure to move abroad. On the other hand, such entrepreneurs can identify and pursue opportunities allowing this kind of a move. Carson and Carson (2018, p. 236) claimed that “lifestyle factors and the search for a better and more balanced quality of life” drive foreign entrepreneurship. Therefore, venturing abroad is an opportunity for those who can afford it. The risks for foreigners from developed countries are certainly lower than those for individuals from less-developed countries, who often have few opportunities after migrating to a more-developed economy (Chrysostome, 2010). The motive of craving for a new start due to being dissatisfied with life in the CoO (e.g. the case of the “fallen Icaruses” in Goxe et al., 2022) seems closer to pursuing opportunities than being a last resort.

Most of the reviewed articles described opportunities to be a significant driver for foreigners from developed countries venturing abroad. However, entrepreneurs do not always discover opportunities in advance. There are two kinds of entrepreneurs: those who already know the opportunities available in a CoR and those who actively seek opportunities abroad (Kumpikaitė-Valiūnienė et al., 2021). When identifying opportunities, a new environment and society surround foreigners staying in CoRs. Embeddedness in networks affects opportunity discovery and enactment (Engelen, 2001). Like ethnic entrepreneurs (Aldrich & Waldinger, 1990), many foreign entrepreneurs first move within co-ethnic communities (e.g. Lassalle & McElwee, 2016; Yang et al., 2011). As a result, they seize co-ethnic opportunities that do not exist in their CoOs (Lundberg & Rehnfors, 2018). Such opportunities target co-ethnics on-site or leverage products and processes from entrepreneurs’ CoOs (Storti, 2014). Therefore, regarding the pursuit of ethnic opportunities, foreign entrepreneurs from developed countries show similar motivations to those encountered in the migrant entrepreneurship literature (e.g. Aldrich & Waldinger, 1990; Kloosterman, 2010).

However, most studies have emphasised market opportunities (e.g. Heidenreich et al., 2015) and location-specific aspects as active drivers of foreign venturing. Favourable institutional conditions attract entrepreneurs (e.g. Elo et al., 2019; March-Chordà et al., 2021). For example, European entrepreneurs looking to start high-tech startups seek opportunities in foreign markets that offer attractive entrepreneurial ecosystems – for example, as can be found in Silicon Valley (March-Chordà et al., 2021). Therefore, they leverage foreign markets and geographies to obtain a competitive advantage (De Cock et al., 2021) and purposefully draw on resources suitable for exploiting foreign market opportunities (Almor & Yeheskel, 2013; March-Chordà et al., 2021).

Moreover, institutional voids create opportunities for those eager to take on the challenge of working in an institutionally unstable environment (Elo et al., 2019). Contrary to the logic that a weak institutional environment harms entrepreneurship (Robinson & Acemoglu, 2012; Wright et al., 2005), institutional voids can also be beneficial (Ensign & Robinson, 2011). Unstable and challenging environmental factors create unique opportunities for those who dare to found a startup in these environments (e.g. Heidenreich et al., 2015). Therefore, such institutional environments allow entrepreneurs to leverage skills, networks and resources to obtain an advantage in pursuing opportunities (Abd Hamid & Everett, 2021; McHenry & Welch, 2018).

In sum, the motivational drivers of foreign entrepreneurs originating from developed countries are external factors that depend on the country context (e.g. Kumpikaitė-Valiūnienė et al., 2021) paired with internal aspects related to entrepreneurs’ personal contexts. However, opportunity-driven pull factors outweigh push factors, which primarily stem from the desire or need to stay in the CoR. Nevertheless, independently of the motivational drivers, entrepreneurial opportunities influence foreign entrepreneurs’ embeddedness in CoRs (e.g. Hudnut & DeTienne, 2010).

### Embeddedness

Studies of migrant entrepreneurs in advanced economies show that opportunity structures and the embeddedness of foreign entrepreneurs are closely related factors (Kloosterman & Rath, 2010; Waldinger et al., 1990). Considering the entrepreneur as an agent and the environment as the social context, ergo the structures the entrepreneurs are moving in (Giddens, 1979), embeddedness abroad depends on two factors. First, an entrepreneurs’ inner attitudes determine how they integrate into CoRs – that is, with whom they associate and how they understand the new local environment. Second, the social structures and mechanisms related to the entrepreneurial process (Jack & Anderson, 2002) ease or complicate the integration process and impact the embeddedness of entrepreneurs.

Embeddedness is dynamic and develops “progressively, through multiple and sequential professional experiences in the host country” (Gurău et al., 2020, p. 10). For example, prior stays and expatriate assignments (Lundberg & Rehnfors, 2018; Selmer et al., 2018) in the CoR provide entrepreneurs with networks and cultural experiences. Our framework identifies four levels of embeddedness in a CoR. The first level involves the entrepreneurs’ community (Goxe et al., 2022), a familiar cultural space that offers access to networks and resources. It serves as the first step in embedding oneself within a CoR (Lassalle et al., 2020). The second level refers to the co-ethnic society within the CoR, which functions as an enabler and a catalyst for foreign venturing (e.g. Abd Hamid & Everett, 2021; Eimermann & Kordel, 2018; Shin, 2014). The third level addresses the host-country society. Entrepreneurs have to embed themselves into the host-country society to overcome institutional challenges (McHenry & Welch, 2018) and enter mainstream markets (Ensign & Robinson, 2011). Finally, only after entering mainstream markets, a global expansion seems possible (De Cock et al., 2021).

Overall, entrepreneurs’ motivations for entering foreign societies matter. Entrepreneurs’ reasons for staying in particular countries influence their degree of embeddedness. As outlined before, some foreigners become entrepreneurs out of the necessity to stay in a CoR. Conversely, those who pursue opportunities have no intention of staying in a CoR permanently but establish ventures to exploit opportunities (Almor & Yeheskel, 2013; McHenry & Welch, 2018).

Entrepreneurs can move across the levels to become more deeply embedded in a CoR (Lassalle et al., 2020) – for example, by grasping local business habits (Ngoma, 2016). At first, many foreign entrepreneurs rely on their co-national communities for support and easier access to resources (Ensign & Robinson, 2011; Thomas & Ong, 2015). Then, entrepreneurs start developing co-ethnic networks and strengthening social ties (Knight, 2015) with host-country nationals to eventually enter mainstream markets (Ensign & Robinson, 2011).

However, some entrepreneurs intentionally adhere to chosen communities (e.g. Goxe et al., 2022) – for instance, those from their CoOs (e.g. Abd Hamid & Everett, 2021) – and do not embed themselves locally. Therefore, the movement from the self-centred to the global level does not apply to all foreigners. Carson and Carson (2018) found that such so-called “international enclaves” purposefully exclude themselves from the local society and move somewhere between their co-national communities and co-ethnic societies, disregarding local interests. Surprisingly, not all co-ethnic entrepreneurs trust their communities, even though relying on networks and resources is a common practice (Lassalle & McElwee, 2016).

One clear difference between migrant entrepreneurs going to developed countries and entrepreneurs coming from developed countries is the desire to immigrate and settle in the host country in the long term. The necessity-driven entrepreneurs described above desire to stay in the host country temporarily but do not have permanent plans to do so (Elo et al., 2019). Similarly, lifestyle entrepreneurs are susceptible to lifestyle changes and may pursue other lifestyles in other countries in the future. Therefore, the urge to assimilate into a CoR and to establish firm ties with the local society is limited (Eimermann & Kordel, 2018). For foreigners from developed countries, it seems uncertain whether it is worth taking the arduous path of integrating into a foreign environment when the stay duration in the CoR is limited.

There is no doubt that deep local roots are a prerequisite for fully leveraging local resources and building a company capable of operating in local mainstream markets and potentially achieving global reach. A strong bond with the host country strengthens entrepreneurial capacity and should, therefore, be envisaged from early on (Selmer et al., 2018). Moreover, companies targeting local customers (Tucker & Croom, 2021) – for instance, through social entrepreneurship – need to fully integrate into the CoR. Otherwise, fully understanding the foreign market is difficult (Hudnut & DeTienne, 2010). In fact, foreignness can become a strength, as it can help an entrepreneur leverage the foreign community’s resources and networks in the CoR and the CoO. Logically, the social and economic impact of the entrepreneur on the host country also depends on the degree of embeddedness. The larger the target market, the greater the economic and social impact that an entrepreneur can create.

### Impact

Entrepreneurship is an essential pillar of the economy that creates economic and social impacts (Schumpeter & Backhaus, 2003). However, the type of business and its embeddedness in a local society can make all the difference between a niche business and a company with tremendous growth potential (Saxenian, 2007). Moreover, embeddedness varies depending on entrepreneurs’ intentions of becoming locally embedded (e.g. Goxe et al., 2022), the chosen level of embeddedness (Lassalle et al., 2020) and the ability to become embedded (e.g. Abd Hamid & Everett, 2021; Lassalle & McElwee, 2016). Therefore, our framework categorises entrepreneurs venturing abroad into four archetypes along a continuum from low to high social and economic impacts (see Fig. 4). These four archetypes of international entrepreneurs are *xenophile social entrepreneur*, *glocalised lifestyle entrepreneur*, *glocalised opportunity seeker* and *international opportunity seeker*.

The social impact of foreign entrepreneurship depends on their integration and embeddedness in local structures. It seems improbable that an entrepreneur with little to no contact with local customers or employees could significantly impact society. Nevertheless, such an entrepreneur can generate a robust economic impact. In particular, entrepreneurs who engage with limited communities or adopt international business models can create sustainably profitable businesses that indirectly benefit host countries by bringing in scarce resources (Almor & Yeheskel, 2013). Naturally, the associated foreign entrepreneurial investments and the resulting impacts are low compared to those of multinational enterprises at least initially (Almor & Yeheskel, 2013). However, the future potential of some of these ventures is promising (De Cock et al., 2021). Looking at the foreign entrepreneurial impact in advanced economies (Kloosterman & Rath, 2001, 2010), specifically in the case of Silicon Valley (Saxenian, 2007), the belief in foreign entrepreneurship and its potential economic dimensions is well-grounded. Therefore, robust economic impact can later on promote social impact.

#### Xenophile social entrepreneurs

Tucker and Croom (2021) examined the phenomenon of venturing for foreigners, specifically the xenophile aspect of entrepreneurship. Foreigners venturing abroad for social reasons with a low economic impact fit this idea. One could also argue that social entrepreneurship addressing social problems are included in this archetype. However, engaging in social entrepreneurship in a foreign CoR is a phenomenon that emphasises operating a social business for other people coming from another CoO. Therefore, xenophile entrepreneurs from developed countries are less motivated by pursuing opportunities that increase their economic gains and more motivated by social impacts for the underprivileged (Marshall, 2011).

#### Glocalised lifestyle entrepreneur

In contrast to entrepreneurs who act based on social motivation, glocalised lifestyle entrepreneurs are not particularly successful in economic terms, nor do they contribute to improving social structures (Carson & Carson, 2018). The scope of their entrepreneurial activity hardly reaches beyond their extended community (Eimermann & Kordel, 2018). For this archetype, entrepreneurship is arguably more of a means to an end, with no grand intentions. Instead of becoming entrepreneurs out of necessity, the persons belonging to this archetype pursue entrepreneurship to lead their preferred lifestyles in the host country. (McHenry & Welch, 2018).

#### Glocalised opportunity seeker

Glocalised opportunity seekers are firmly anchored in their CoRs and purposefully realise entrepreneurial opportunities. These entrepreneurs operate beyond their communities and co-ethnic networks (e.g. Dillon et al., 2020; Goxe et al., 2022; Selmer et al., 2018). Many such foreign entrepreneurs have work experience as expatriates (e.g. Connelly, 2010; McHenry & Welch, 2018; Selmer et al., 2018) and have professional social networks in their CoOs and CoRs (Ngoma, 2016). Therefore, they combine the strength of using the resources of both the CoO and the CoR to mitigate the challenges associated with venturing abroad (e.g. Gurău et al., 2020). However, these entrepreneurs do not achieve a deep enough local anchoring to run established large companies with unique international selling points or to seize the next significant opportunity (e.g. Almor & Yeheskel, 2013). Nevertheless, they are economically prosperous and make a non-negligible contribution to economic development.

#### International opportunity seeker

International opportunity seekers proactively go to host countries to take advantage of existing structures and to make the best possible use of entrepreneurial opportunities (De Cock et al., 2021; March-Chordà et al., 2021). These entrepreneurs combine resources from their home countries with those from the host country and orient themselves globally from the very beginning of the venture. Therefore, their local embeddedness is deep, and their economic contributions have enormous potential (Saxenian et al., 2002).

Table 6 further illustrates the different archetypes of foreign entrepreneurs from developed countries by providing vignettes derived from the article data.

**Table 6:** Archetypes of foreign entrepreneurs according to their impacts on CoRs

**Category**	**Archetype**	**Examples from selected articles**
Xenophile social entrepreneur	Educated in developed countries and raised in wealthy environments, these entrepreneurs aim to set up companies to increase the living standards in the less-developed world. They have been in contact with foreigners from less-developed countries and have visited such countries frequently. Being aware of their privileges, such entrepreneurs want to help host societies improve education, increase health or achieve other ends aligned with the United Nations Sustainable Development Goals. The entrepreneur is intrinsically motivated to do good in the world rather than live a prosperous life. Thus, the xenophile social entrepreneur strives for recognition for achievements in improving others’ lives rather than for economic benefits	“[They] come from nonprofit organizations with a small expatriate workforce, or possibly are on their own. These organizations (or individuals) may be new to the international arena and are likely to develop policies and procedures to address the battery of issues they face in their new environment. Expatriate assignments for this group are more ideologically and may be a reward in their own right rather than a means to a career end.” (Connelly, 2010:44) “[We] focus on individual experiences before the social venture is formed, with the recognition that some individuals interact with foreigners before social venture opportunities are recognized, explored, or exploited. We introduce xenophilia as a trait that some people have and employ through entrepreneurial practices.” (Tucker & Croom, 2021:e00217)
Glocalised lifestyle entrepreneur	The glocalised lifestyle entrepreneur comes from Western Europe and is either a discoverer in their late 20 s or a middle-aged escapist running away from the CoO society. They have received a good education in their CoOs and have gained some work experience in their CoOs and CoRs. Knowing the advantages of their CoRs, these entrepreneurs seek particular lifestyles there. Venturing abroad is a means to an end that enables entrepreneur to stay in the CoR, allowing them to achieve the desired way of living. However, the business is barely scalable and leverages some cross-border resources	“Lifestyle migrants continuously pursue practices based on their hobbies or special interests to realise their desired way of life” (Eimermann & Kordel, 2018:2) “All participants identified lifestyle factors and the search for a better and more balanced quality of life as the major reasons for their relocations. They mostly originated from urban or densely populated areas, and cited common counter-urban migration motives as the main reasons for moving (Carson & Carson, 2018:13) “The second group we designated as expatriate entrepreneurs. These individuals had established their own business, either as sole owners or in conjunction with a local partner (usually their Vietnamese spouse)” (McHenry & Welch, 2018:94) ”Five had started their own companies of which they were sole or part owners: Three were construction-related firms, one a pharmaceutical distribution company, and another established an international management consultancy … Their self-image and lifestyle were described as being more like the traditional expatriate; but there was consensus that, in terms of status, employment conditions and income, they were similar to immigrants or locals.” (McHenry & Welch, 2018:95)
Glocalised opportunity seeker	Coming from a developed country, the glocalised opportunity seeker is well-educated. However, the necessary knowledge for venturing abroad comes from prior stays in the CoR. They either worked as an expatriate in the CoR or have some links to the co-ethnic society. Therefore, the entrepreneur has an established network and access to resources, helping their business flourish from early on. In addition, the familiar institutional setting of the CoR helps identify opportunities and mitigate challenges. The glocalised opportunity seeker intends to continue the stay for the time being. However, long-term orientation is not necessarily related to the CoR. Therefore, the entrepreneur aims to include host-country nationals in the business, allowing them to expand to local markets and establish a stable business	“Almost all the entrepreneurs had some experience of other countries through studies, travel, or work. Also, they had either some … or extensive … work experience in Asia prior to starting their businesses in Hong Kong that had resulted in foreign business knowledge as well as institutional knowledge.” (Lundberg & Rehnfors, 2018:161) “Expat-preneurs were older, had higher positions, had spent a longer time in their current job in the host location, had been expatriates longer, and had been in the host location for a longer time…. [The] path to becoming an expat-preneur (whether pre-departure or transitioned) is influenced significantly by temporality … Personal characteristics such as maturity and deep experience living and working abroad may be common traits among expat-preneurs.“ (Selmer et al., 2018:143f) “Integration in a transnational professional network provides them with opportunities to develop their social and cultural capital in [the CoR], which later facilitate the accumulation of economic (e.g., wealth) and symbolic (e.g., status, recognition) capital.” (Gurău et al., 2020:10)
International opportunity seeker	These entrepreneurs have an open mindset, are well-educated and have previous international experience. In addition, they are multilingual, business experienced and culturally skilled. Thus, they have established international networks that they can leverage for support and introductions. They actively seek opportunities globally and exploit them where the environment fits best. They work together with host-country nationals and draw on experts worldwide	“U.S. educated immigrants sharing with partners across the globe similar entrepreneurial values and practices, capitalizing on their experience and the support of professional networks to identify and exploit international opportunities wherever they are.” (Goxe et al., 2022) “The sojourner, being an outsider from a developed country, brings along many resources and capabilities that do not necessarily exist in the host country. The sojourner brings along know-how, international contacts and experience, relations with venture capital, entrepreneurial experience and so on. … [He] seems to be new as it is based on a person who has a variety of different resources and capabilities, created in developed countries, which he exploits in emerging economies.” (Almor & Yeheskel, 2013:366)

## Discussion

Our study explored, at the individual level, what drives foreign entrepreneurial activity from developed economies to other developed, emerging or developing economies and with what consequences. After systematically reviewing the literature, we introduced a unifying framework for the foreign entrepreneurial journey, which includes contextual influences, motivations for venturing abroad, embeddedness in CoRs, and the resulting social and economic impacts.

### Motivation, embeddedness and impact condition one another

Throughout the entrepreneurial journey, the institutional environment in a CoR influences entrepreneurial activities by facilitating or restricting them. Therefore, the country context is highly significant when investigating foreign entrepreneurial activities (Welter, 2011; Wright et al., 2005). However, the entrepreneurial journey is complex and can only be properly understood by also considering the entrepreneur’s personal context. Prior studies have indicated that migrant entrepreneurs who emigrate to developed countries mainly venture out of necessity (Block & Wagner, 2010; Chrysostome, 2010) – for example, due to the inability to enter the local labour market (Zhou, 2004). Foreign entrepreneurs from developed countries, in turn, are much more driven by their personal abilities and desires (e.g. Almor & Yeheskel, 2013; Goxe et al., 2022); therefore, they can be described as “opportunity immigrant entrepreneurs” rather than “necessity immigrant entrepreneurs” (Chrysostome, 2010, p. 139). As a result, such entrepreneurs usually do not intend to stay permanently in a CoR. Instead, they want to pursue specific opportunities or stay for personal reasons, often based on familiar motivations (McHenry & Welch, 2018). Thus, the personal context determines how such entrepreneurs embed themselves in CoRs and, eventually, what impacts they create.

**Fig. 5:**
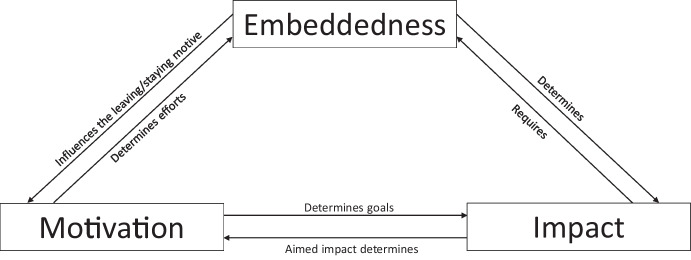
The interrelations of motivation, embeddedness and impact

At first, personal motivation seems to be the starting point of the entrepreneurial journey, determining the efforts undertaken for deeper embeddedness in a CoR, which further influences the impact that an entrepreneur can have. However, the relationship between motivation, embeddedness and impact is linear and direct. In other words, it is not simply motivation and embeddedness that condition the impacts that entrepreneurs can produce; instead, all three aspects determine and condition one another. Figure 5 outlines the interrelations of the three aspects.

Entrepreneurs’ desired impact creation requires a certain depth of embeddedness in a CoR (Ensign & Robinson, 2011). Furthermore, a clearly stated impact goal affects the entrepreneurial motivation – for instance, when venturing for social reasons (Marshall, 2011; Tucker & Croom, 2021). Moreover, motivation and embeddedness partly account for the differences between entrepreneurs from developed and developing economies. For example, when the intention to stay in a CoR is time-bound, the motivation to make efforts to become locally embedded likely is lower (Elo et al., 2019), which prevents entrepreneurs from reaching their full impact potential (Goxe et al., 2022).

Furthermore, embeddedness affects entrepreneurial motivation. For instance, deeply embedded foreigners with staying motives can choose the entrepreneurial path to remain in a CoR (McHenry & Welch, 2018). There is a connection between the impact and the motivation. The motivation to achieve a desired impact leads to perseverance in achieving the set target. Therefore, motivation indirectly affects the efforts entrepreneurs are willing to make to reach their impact goals. For example, it is possible increase one’s local embeddedness to increase one’s resources (Knight, 2015; Lassalle et al., 2020). Therefore, understanding the threefold relation of motivation, embeddedness and impact is essential for comprehending foreign entrepreneurs.

### Categorising foreign entrepreneurs according to their impact

As mentioned initially, there are many categories and terms of foreigners venturing abroad: sojourners (Almor & Yeheskel, 2013), expat-preneurs (Selmer et al., 2018; Vance et al., 2016) and lifestyle entrepreneurs (Carson & Carson, 2018), to name a few. Further, more compelling research fields have evolved, such as diaspora entrepreneurship (Elo, 2016), transnational entrepreneurship (Drori et al., 2009), ethnic entrepreneurship (Aldrich & Waldinger, 1990) and migrant entrepreneurship (Baycan-Levent & Nijkamp, 2009). However, we do not sort the entrepreneurs according to their ethnicity or call them migrants because their intentions to stay in a CoR are unclear. Furthermore, some operate transnational businesses (e.g. Heidenreich et al., 2015; Lundberg & Rehnfors, 2018), while others work at the local level (e.g. Carson & Carson, 2018). In addition, their entrepreneurial motivations and intentions differ, as some entrepreneurs start as expatriates in a CoR; others, however, actively seek opportunities in emerging economies and act as sojourners or move to markets where they see the highest potential (Almor & Yeheskel, 2013; Chrysostome, 2010; Gurău et al., 2020).

Therefore, coming back to the “South to North” analogy of entrepreneurs travelling to more-developed economies (Kumpikaitė-Valiūnienė et al., 2021), entrepreneurs from developed economies are globally oriented entrepreneurs who seek to exploit international opportunities where they encounter them. Arguably, they are “elite diasporans with developed skills and numerous alternatives for their career and livelihood” (Elo, 2016, p. 123). Although their entrepreneurial motivations and personal contexts differ, one might argue that such foreign entrepreneurs from developed countries, on average, also operate within the institutional arena they are familiar with, for example, they engage with co-ethnics and the expat community (Lundberg & Rehnfors, 2018). They actively extend their social capital by mobilising networks and resources when needed (Goxe et al., 2022; Ngoma, 2016). Thus, their resources enable them to use the dynamics of embeddedness to their advantage, differently from necessity-driven immigrant entrepreneurs (Chrysostome, 2010).

Nonetheless, foreigners from developed countries venturing abroad practice entrepreneurship and, thus, benefit economic growth and overall prosperity of the host country (Robinson & Acemoglu, 2012). Furthermore, “brain circulation” (Saxenian, 2007) significantly drives innovation, technology transfer and economic development. Therefore, the introduced framework has proposed categorising entrepreneurs according to their impact on CoRs by looking at social and economic dimensions. Furthermore, clustering entrepreneurs according to their impact improves our shared understanding of the motivations and outcomes of foreign entrepreneurship.

### Policymakers benefit from furthering foreign entrepreneurship

Brain circulation positively impacts the development of innovation and entrepreneurial landscapes (Filatotchev et al., 2011; Saxenian, 2007). Venturing abroad can be associated with positively contributing to a host country, regardless of whether it is a developed or less-developed economy. Moreover, the experience and knowledge acquired in a CoR likely benefit the CoO at a particular stage – for example, when entrepreneurs return to their CoOs (Gruenhagen et al., 2020; Wright et al., 2008). As a result of venturing abroad, entrepreneurs bring back the human and social capital and the networks (Light et al., 2017) they acquired or developed in the CoRs. Drori et al. (2009, p. 1006) called them “scientists and engineers returning to their home countries to start up a new venture.” Looking at the characteristics of *international opportunity seekers*, it is likely that such entrepreneurial individuals contribute to both their CoOs and CoRs. Therefore, governments in CoRs and CoOs should support foreign entrepreneurship to maximise its social and economic benefits.

### Implications for foreign entrepreneurs

Founding and operating a company is a demanding and long-term endeavour, even more so when venturing into a foreign country with unfamiliar institutional dimensions (Welter, 2011). Entrepreneurs’ personal contexts and life situations impact the whole entrepreneurial journey, and entrepreneurs have to act upon the identified opportunities deliberately. Therefore, entrepreneurs should be aware of the opportunities available to their businesses. For example, if the goal is to pursue a desired lifestyle (Carson & Carson, 2018), the company is more of a means to an end.

However, if the aim is to achieve a social goal, such as minimising the social grievances of the host population (Tucker & Croom, 2021), close cooperation with local institutions is necessary to either use them or change them for the better (Bruton et al., 2010). For example, international opportunity seekers bring their globally acquired resources and thus seize opportunities in the best possible way; glocalised lifestyle entrepreneurs, however, prioritise their desired ways of living. Therefore, to minimise uncertainty, entrepreneurs must define their goals for venturing abroad early on and be clear regarding the motivations and consequences.

Our study has shown that the degree of embeddedness within local society affects the economic and social impact that an entrepreneur eventually creates. Thus, vision-driven entrepreneurs aiming to enter mainstream markets must increase their social and cultural capital (Bourdieu, 2005; Goxe et al., 2022) to avoid being limited to niche markets (Ensign & Robinson, 2011). Active participation in and interaction with the CoR society is essential (Lassalle & Scott, 2018). Moreover, entrepreneurs can turn their foreignness into an advantage by drawing on a dual habitus (Goxe et al., 2022; Gurău et al., 2020) to overcome institutional voids (Drori et al., 2009).

### Limitations and future research agenda

Some limitations of our study point to interesting opportunities for future research. Furthermore, reviewing the literature systematically helps identify research gaps that need to be bridged in the near future.

First, the review has provided an overarching look at entrepreneurs from developed countries. However, it was beyond our scope to analyse each component of the introduced framework in significant detail. Therefore, it would be worthwhile to investigate, for example, how the dynamics of embeddedness unfold for foreign entrepreneurs from developed countries compared to foreign entrepreneurs from less-developed countries venturing abroad. Furthermore, the study did not include geographical comparisons. The framework is general but lacks a focus on country-specific aspects, such as cultural distance or country-specific characteristics. Thus, investigating foreign entrepreneurship in regional areas and identifying area-specific attributes would open up future research avenues that would, in turn, help us understand the phenomenon of foreign entrepreneurship better while deriving more specific practical implications.

Second, theoretical saturation is far from being reached, and the theoretical lens needs further sharpening. Very few articles included in our review draw on a theoretical framework. As the articles adopt different overarching definitions of foreign entrepreneurship, such as ethnic entrepreneurship (e.g. Yang et al., 2011), no unifying principle is apparent. Therefore, the field would benefit from theoretical explanations based on the introduced framework to understand what drives foreign entrepreneurship and with what consequences. Explanations of the motivations and consequences shed light on how foreign entrepreneurial activity could be steered towards more significant social and economic impacts.

Third, foreign entrepreneurship offers promising future research avenues that could provide a better overall understanding of foreigners from developed countries venturing abroad. Expatriates operate multinational companies globally. Therefore, it is likely that the tendency of entrepreneurs from developed countries venturing into their already familiar CoRs will increase (Selmer et al., 2018; Vance et al., 2016). Moreover, the exogen shock caused by COVID-19 (Polack et al., 2020) increased the number of people working remotely (Ozimek, 2020), encouraging lifestyle-driven employees to move to new CoRs. This extraordinary event has underlined the notion that the cross border talent movement from developed economies is increasing. Thus, lifestyle entrepreneurship will likely increase, and the possibility of qualified and well-resourced individuals becoming opportunity seekers is high. Therefore, further research on this topic is crucial.

Fourth, to achieve a global perspective on the phenomenon, researchers must close geographical gaps. For example, none of the studies examined focused on India, and only one study investigated the African context. Nevertheless, the demographic developments in both places imply that their role in future economic developments will increase. Therefore, entrepreneurship from developed countries to these significant locations has likely already begun. If so, scholars must close this knowledge gap. Moreover, entrepreneurs who have lived abroad for a long time bring back numerous experiences, approaches and innovations (Dai & Liu, 2009; Liu et al., 2010) to their developed home countries. Thus, investigating returnee entrepreneurship from less developed economies to developed economies has excellent research potential.

Fifth, a more quantifiable methodological lens is necessary to better understand the effects and relations of variables that impact foreign entrepreneurs. The populations studied have been comparably small, and it is challenging to obtain a sufficiently large sample for a quantitative study. However, only when scholars overcome this obstacle can the field test new theoretical ideas and concepts to achieve significant knowledge about variables outcomes and dependencies. Qunatifying, for example, the average social and economic impact of a foreigner venturing abroad could be used as an argument for easing institutional challenges and actively supporting foreign entrepreneurship. This hurdle can be overcome primarily through collaboration and cooperation between researchers. Therefore, it is essential to build alliances to create shared access to scarce informants in the future.

Finally, the review shows that quantitative data on the phenomenon is generally scarce. International entrepreneurship research depends on investigating cross-border movements, which leads to challenges in identifying and accessing the population (Davidsson & Wiklund, 2001). Without access to high-quality data, the research field will not be able to unfold its full potential. An accurate recording of founder identities at the national state level or any other governing institution would be a good starting point to allow for interesting future research.

## Conclusion

Approximately only one out of seven people live in a developed country. Therefore, entrepreneurial individuals from developed countries with a flair for seeking opportunities can play a unique role in driving technology transfer and creating global innovations. Moreover, impactful entrepreneurial activities positively affect social structures, thus contributing to the development of the countries that entrepreneurs venture to. Our review has shown that the individual context plays an essential role in foreign entrepreneurship.

Entrepreneurs who have had their education in the world's most developed countries can contribute significantly to solving the global challenges ahead. Many opportunities arise outside their economies for a variety of reasons. Opportunities are not limited to a country’s borders, nor are talented entrepreneurs. The most populous countries, such as India and China, and also emerging countries in Africa and South America will become increasingly important economically, ecologically and socially in the coming years. Research on international entrepreneurship has shown that foreign entrepreneurs can contribute greatly to a country's entrepreneurial ecosystem. Furthermore, a connected world depends on migration in various directions that guarantee a steady exchange of ideas, knowledge, and culture. The effects of several decades of globalization bring along a new generation of entrepreneurs actively seeking global opportunities.

Therefore, it is of great academic and practical interest to better understand the foreign entrepreneurial process. The academic discourse would benefit from focusing on these individuals to gain more insights into the motivations behind and conditions of the entrepreneurial process, which are essential for establishing enterprises in foreign markets. This literature review is a starting point that offers an omnibus perspective on foreign entrepreneurship and highlights directions for future research.

## Data Availability

Available upon request.

## Appendix 1. Fortune 500 companies founded by immigrant entrepreneurs from developed countries (Center for American Entrepreneurship, 2017)

**Table:**

**Company**	**Immigration status**	**Founder**	**Country of Origin**
AT&T	Immigrant	Alexander Graham Bell	Scotland
AmerisourceBergen	Immigrant	Lucien Brunswig	France
General Electric	Immigrant	Elihu Thomson, Thomas Edison (child)	England, Canada
Verizon Communications	Immigrant	Alexander Graham Bell	Scotland
Alphabet	Immigrant	Sergey Brin	Russia
Comcast	Immigrant	Daniel Aaron	Germany
Procter & Gamble	Immigrant	William Procter, James Gamble	England, Ireland
PepsiCo	Immigrant	William Loft, Charles Guth (child)	England, Germany
Pfizer	Immigrant	Charles Pfizer, Charles Erhart	Germany
Dow Chemical	Immigrant	Herbert Henry Dow	Canada
Cigna	Immigrant	John M. Nesbitt	Ireland
Honeywell International	Immigrant	Albert Butz	Switzerland
Goldman Sachs Group	Immigrant	Marcus Goldman	Germany
TIAA	Immigrant	Andrew Carnegie	Scotland
Exelon	Immigrant	Samuel Insull	England
General Dynamics	Immigrant	John Philip Holland	Ireland
Capital One Financial	Immigrant	Nigel Morris	England
Philip Morris International	Immigrant	Gustave Eckmeyer	Germany
Mondelez International	Immigrant	James L. Kraft	Canada
DuPont	Immigrant	E.I. du Pont	France
Qualcomm	Immigrant	Andrew Viterbi	Italy
U.S. Bancorp	Immigrant	Donald Macleay	Scotland
International Paper	Immigrant	Hugh Chisholm	Canada
Emerson Electric	Immigrant	Charles and Alexander Meston	Scotland
Altria Group	Immigrant	Gustave Eckmeyer	Germany
Fluor	Immigrant	John Simon Fluor Sr	Switzerland
Kohl's	Immigrant	Maxwell Kohl	Poland
PG&E Corp	Immigrant	George Roe	Canada
Bank of New York Mellon Corp	Immigrant	Thomas Mellon	Northern Ireland
Colgate-Palmolive	Immigrant	William Colgate	England
PPG Industries	Immigrant	John Pitcairn Jr	Scotland
Synchrony Financial	Immigrant	Elihu Thomson, Thomas Edison (child)	England, Canada
Nordstrom	Immigrant	John W. Nordstrom	Sweden
Synnex	Immigrant	Robert T. "Bob" Huang	Taiwan
Texas Instruments	Immigrant	Cecil Green, J. Erik Jonsson (child), Patrick Haggerty (child)	England, Sweden, Norway
Guardian Life Ins. Co. of America	Immigrant	Hugo Wesendonck	Germany
Principal Financial	Immigrant	Archibald Graham "A.G." McIlwaine	Ireland
Biogen	Immigrant	Charles Weissmann, Ivor Royston	Hungary and Switzerland, England
Las Vegas Sands	Immigrant	Jake Freedman	Russia
Celgene	Immigrant	Sol Barer	Germany
CSX	Immigrant	Cyrus S. Eaton	Canada
Entergy	Immigrant	Elihu Thomson, Thomas Edison (child)	England, Canada
PayPal Holdings	Immigrant	Max Levchin, Luke Nosek, Peter Thiel	Russia, Poland, Germany
Praxair	Immigrant	Carl von Linde	Germany
United States Steel	Immigrant	Andrew Carnegie	Scotland
Thrivent Financial for Lutherans	Immigrant	Albert Voecks	Germany
Genworth Financial	Immigrant	Archibald Graham "A.G." McIlwaine	Ireland
Veritiv	Immigrant	Hugh Chisholm	Canada
News Corp	Immigrant	Rupert Murdoch	Australia
Crown Holdings	Immigrant	William Painter	Ireland
Global Partners	Immigrant	Abraham Slifka	Poland
PVH	Immigrant	Moses and Endel Phillips, John Van Heusen	Poland, Holland
Weyerhaeuser	Immigrant	Frederick Weyerhaeuser	Germany
Steel Dynamics	Immigrant	Mark Millett	England
CenterPoint Energy	Immigrant	Thomas W. House	England
National Oilwell Varco	Immigrant	Baldwin Reinhold, Walter Abegg	Switzerland
Tesla	Immigrant	Elon Musk	South Africa
Harman International Industries	Immigrant	Sidney Harman, Bernard Kardon	Canada, Russia and Lithuania
Nvidia	Immigrant	Jensen Huang	Taiwan
R.R. Donnelley & Sons	Immigrant	Richard R. Donnelley	Canada
Quintiles IMS Holdings	Immigrant	Dennis Gillings	England
Sealed Air	Immigrant	Marc A. Chavannes	Switzerland
Targa Resources	Immigrant	Eric Warburg	Germany
Expeditors International of Washington	Immigrant	Peter Rose, James Wang	Canada, Taiwan
Regions Financial	Immigrant	Charles Linn	Finland
Alaska Air Group	Immigrant	Charles H. Ruttan	Canada
NetApp	Immigrant	James Lau	Hong Kong
Celanese	Immigrant	Camille and Henry Dreyfus	Switzerland
Kelly Services	Immigrant	William Russell Kelly	Canada
CH2M Hill	Immigrant	Fred Merryfield	England
ABM Industries	Child	Morris Rosenberg	Poland

## Appendix 2. PRISMA checklist provided by Page et al. (2021)

**Table:**

**Section and Topic**	**Item #**	**Checklist item**	**Location where item is reported**
**TITLE**			
Title	1	Identify the report as a systematic review	p.1
**ABSTRACT**			
Abstract	2	See the PRISMA 2020 for Abstracts checklist	
**INTRODUCTION**			
Rationale	3	Describe the rationale for the review in the context of existing knowledge	Introduction
Objectives	4	Provide an explicit statement of the objective(s) or question(s) the review addresses	Introduction
**METHODS**			
Eligibility criteria	5	Specify the inclusion and exclusion criteria for the review and how studies were grouped for the syntheses	2.3 (p.6f.); 2.4 (p.8f.)
Information sources	6	Specify all databases, registers, websites, organizations, reference lists and other sources searched or consulted to identify studies. Specify the date when each source was last searched or consulted	2.2 (p.5)
Search strategy	7	Present the full search strategies for all databases, registers and websites, including any filters and limits used	2.1 (p. 4); Table 1; 2.2 (p.5)
Selection process	8	Specify the methods used to decide whether a study met the inclusion criteria of the review, including how many reviewers screened each record and each report retrieved, whether they worked independently, and if applicable, details of automation tools used in the process	2.4 (p.9)
Data collection process	9	Specify the methods used to collect data from reports, including how many reviewers collected data from each report, whether they worked independently, any processes for obtaining or confirming data from study investigators, and if applicable, details of automation tools used in the process	2.5 (p.10) & Table 3
Data items	10a	List and define all outcomes for which data were sought. Specify whether all results that were compatible with each outcome domain in each study were sought (e.g. for all measures, time points, analyses), and if not, the methods used to decide which results to collect	2.5 & Table 3
	10b	List and define all other variables for which data were sought (e.g. participant and intervention characteristics, funding sources). Describe any assumptions made about any missing or unclear information	2.5 & Table 3
Study risk of bias assessment	11	Specify the methods used to assess risk of bias in the included studies, including details of the tool(s) used, how many reviewers assessed each study and whether they worked independently, and if applicable, details of automation tools used in the process	./
Effect measures	12	Specify for each outcome the effect measure(s) (e.g. risk ratio, mean difference) used in the synthesis or presentation of results	Not applicable
Synthesis methods	13a	Describe the processes used to decide which studies were eligible for each synthesis (e.g. tabulating the study intervention characteristics and comparing against the planned groups for each synthesis (item #5))	Not applicable
	13b	Describe any methods required to prepare the data for presentation or synthesis, such as handling of missing summary statistics, or data conversions	Not applicable
	13c	Describe any methods used to tabulate or visually display results of individual studies and syntheses	p. 11; Table 3
	13d	Describe any methods used to synthesize results and provide a rationale for the choice(s). If meta-analysis was performed, describe the model(s), method(s) to identify the presence and extent of statistical heterogeneity, and software package(s) used	Not applicable
	13e	Describe any methods used to explore possible causes of heterogeneity among study results (e.g. subgroup analysis, meta-regression)	Not applicable
	13f	Describe any sensitivity analyses conducted to assess robustness of the synthesized results	Not applicable
Reporting bias assessment	14	Describe any methods used to assess risk of bias due to missing results in a synthesis (arising from reporting biases)	./.
Certainty assessment	15	Describe any methods used to assess certainty (or confidence) in the body of evidence for an outcome	./.
**RESULTS**			
Study selection	16a	Describe the results of the search and selection process, from the number of records identified in the search to the number of studies included in the review, ideally using a flow diagram	2.4 (p.10), Fig. 1
	16b	Cite studies that might appear to meet the inclusion criteria, but which were excluded, and explain why they were excluded	2.4 (p.10)
Study characteristics	17	Cite each included study and present its characteristics	p. 11; Table 3
Risk of bias in studies	18	Present assessments of risk of bias for each included study	./.
Results of individual studies	19	For all outcomes, present, for each study: (a) summary statistics for each group (where appropriate) and (b) an effect estimate and its precision (e.g. confidence/credible interval), ideally using structured tables or plots	Not applicable
Results of syntheses	20a	For each synthesis, briefly summarise the characteristics and risk of bias among contributing studies	Not applicable
	20b	Present results of all statistical syntheses conducted. If meta-analysis was done, present for each the summary estimate and its precision (e.g. confidence/credible interval) and measures of statistical heterogeneity. If comparing groups, describe the direction of the effect	Not applicable
	20c	Present results of all investigations of possible causes of heterogeneity among study results	Not applicable
	20d	Present results of all sensitivity analyses conducted to assess the robustness of the synthesized results	Not applicable
Reporting biases	21	Present assessments of risk of bias due to missing results (arising from reporting biases) for each synthesis assessed	Not applicable
Certainty of evidence	22	Present assessments of certainty (or confidence) in the body of evidence for each outcome assessed	Not applicable
**DISCUSSION**			
Discussion	23a	Provide a general interpretation of the results in the context of other evidence	Chapter 4 (p. 28 ff.)
	23b	Discuss any limitations of the evidence included in the review	4.5
	23c	Discuss any limitations of the review processes used	4.5 (p. 33)
	23d	Discuss implications of the results for practice, policy, and future research	4.3/4.4 (p.32 ff.)
**OTHER INFORMATION**			
Registration and protocol	24a	Provide registration information for the review, including register name and registration number, or state that the review was not registered	
	24b	Indicate where the review protocol can be accessed, or state that a protocol was not prepared	On request
	24c	Describe and explain any amendments to information provided at registration or in the protocol	
Support	25	Describe sources of financial or non-financial support for the review, and the role of the funders or sponsors in the review	Title page
Competing interests	26	Declare any competing interests of review authors	Title page
Availability of data, code and other materials	27	Report which of the following are publicly available and where they can be found: template data collection forms; data extracted from included studies; data used for all analyses; analytic code; any other materials used in the review	On request

## Appendix 3. Bibliometric summary statistics of the articles

**Table:**

**Summary description of articles**	**Results**
Timespan	2010:2022
Sources (Journals)	20
Articles	33
Document Average Age	5,64
Average citations per doc	21,91
Total References	2391
Author's Keywords (DE)	137
Authors	70
Single-authored docs	8
Avg. Co-Authors per Doc	2,21
International co-authorships %	42,42
